# Effect of Shallow and Deep SCUBA Dives on Heart Rate Variability

**DOI:** 10.3389/fphys.2018.00110

**Published:** 2018-02-27

**Authors:** Yeonsik Noh, Hugo F. Posada-Quintero, Yan Bai, Joseph White, John P. Florian, Peter R. Brink, Ki H. Chon

**Affiliations:** ^1^Department of Electrical and Computer Engineering, College of Nursing, University of Massachusetts, Amherst, MA, United States; ^2^Department of Biomedical Engineering, University of Connecticut, Storrs, CT, United States; ^3^Department of Biomedical Engineering, Worcester Polytechnic Institute, Worcester, MA, United States; ^4^Department of Physiology and Biophysics, State University of New York at Stony Brook, Stony Brook, NY, United States; ^5^Biomedical Research Department, Navy Experimental Diving Unit, Panama City, FL, United States

**Keywords:** SCUBA, autonomic nervous system, heart rate variability, principal dynamic mode, trimix, nitrox, decompression

## Abstract

Prolonged and high pressure diving may lead to various physiological changes including significant alterations of autonomic nervous system (ANS) activity that may be associated with altered physical performance, decompression sickness, or central nervous system oxygen toxicity. Ideally, researchers could elucidate ANS function before, during, and after dives that are most associated with altered function and adverse outcomes. However, we have a limited understanding of the activities of the ANS especially during deeper prolonged SCUBA diving because there has never been a convenient way to collect physiological data during deep dives. This work is one of the first studies which was able to collect electrocardiogram (ECG) data from SCUBA divers at various depths (33, 66, 99, 150, and 200 ftsw; equivalent to 10.05, 20.10, 30.17, 45.72, and 60.96 m of salt water, respectively) breathing different gas mixtures (air, nitrox and trimix). The aim of this study was to shed light on cardiac ANS behavior during dives, including deep dives. With the aid of dry suits, a Holter monitor that could handle the pressure of a 200 ft. dive, and a novel algorithm that can provide a useful assessment of the ANS from the ECG signal, we investigated the effects of SCUBA dives with different time durations, depths and gas mixtures on the ANS. Principal dynamic mode (PDM) analysis of the ECG, which has been shown to provide accurate separation of the sympathetic and parasympathetic dynamics, was employed to assess the difference of ANS behavior between baseline and diving conditions of varying depths and gas mixtures consisting of air, nitrox and trimix. For all depths and gas mixtures, we found consistent dominance in the parasympathetic activity and a concomitant increase of the parasympathetic dynamics with increasing diving duration and depth. For 33 and 66 ft. dives, we consistently found significant decreases in heart rates (HR) and concomitant increases in parasympathetic activities as estimated via the PDM and root mean square of successive differences (RMSSD) for all time intervals (from the first 5 min to the last 30 min) at the bottom depth when compared to the baseline depth at sea level. The sympathetic dynamics did not change with dive duration or gas mixtures, but at the 150 and 200 ft. dives, we found a significant increase in the sympathetic dynamics in addition to the elevated parasympathetic dynamics when compared to baseline The power spectral density (PSD) measures such as the low frequency (LF), high frequency (HF) and its ratio, and approximate entropy (ApEn) indices were not as consistent when compared to PDM-derived parasympathetic dynamics and RMSSD index.

## Introduction

With the development of the self-contained underwater breathing apparatus (SCUBA), diving became a popular recreational activity as well as a professional pursuit. It is estimated that there are approximately 7 million divers worldwide and 500,000 new divers taking up the activity every year (Levett and Millar, 2008). SCUBA diving exposes individuals to many environmental stresses that are uncommon in everyday life. Prolonged and high pressure diving may lead to various physiological changes including significant alterations of autonomic nervous system (ANS) activity during decompression sickness (Schipke and Pelzer, 2001; Chouchou et al., 2009; Bai, 2011; Gempp et al., 2012; Gempp and Louge, 2013).

It is well known that the diving reflex involving the ANS is stimulated when a face is immersed in water. This reflex results in bradycardia due to increased vagal activity (Gooden, 1994). However, there are complex and sometimes conflicting additional ANS responses in an actual SCUBA dive. During immersion in thermoneutral water, the hydrostatic pressure of the surrounding water balances the hydrostatic pressure within the systemic circulation, and shifts the blood from the lower part of body to the central circulation. In head-out water immersion, it was found that the increase in thoracic blood volume alters regional ANS activity further, thereby contributing to bradycardia, increased stroke volume and cardiac output, reduced muscle sympathetic nerve activity and systemic vascular resistance, and unaltered blood pressure (Mano et al., 1985; Epstein, 1992). Furthermore, the increased partial pressure of oxygen in the SCUBA breathing gas increases cardiac parasympathetic tone and decreases the sympathetic activity to the heart and peripheral circulation (Seals et al., 1991; Lund et al., 1999, 2000; Yamauchi et al., 2002). On the other hand, being immersed in cold water may enhance both sympathetic and parasympathetic activities (Mourot et al., 2007). Additionally, mental stress and proficiency of diving skills are also known to influence the autonomic nervous function (Srámek et al., 2000; Flouris and Scott, 2009). In a SCUBA dive, these factors result in complex interactions to modulate the ANS activity. Hence, monitoring cardiac ANS dynamics may lead to a better understanding of diving physiology and become a potential diagnostic marker of hazards associated with prolonged and deeper diving, ideally becoming a measurable warning mechanism to invoke a modification of the activity or environment prior to an incident.

To our knowledge, there are only a few studies designed to better understand dynamics of the cardiac ANS activity during SCUBA dives (Schipke and Pelzer, 2001; Chouchou et al., 2009). These studies showed dominance of the parasympathetic system during SCUBA diving. However, these studies involved shallow depths (<66 ft.) and did not carefully control dive profiles. Additionally, these studies estimated the ANS activity using power spectral density (PSD) analysis of heart rate (HR) variability (HRV). HRV derived from an electrocardiogram (ECG) signal is a popular non-invasive method for approximating ANS dynamics in both dry and wet environments. The PSD predominantly provides two spectral frequency bands, the low frequency (LF) from 0.04 to 0.15 Hz, and the high frequency (HF) from 0.15 to 0.5 Hz, with the former widely accepted to be mediated by both sympathetic and parasympathetic systems and the latter believed primarily to reflect parasympathetic tone (Task Force of the European Society of Cardiology and the North American Society of Pacing Electrophysiology, 1996). While the PSD method is widely used, its true efficacy as an accurate ANS index is questionable (Task Force of the European Society of Cardiology and the North American Society of Pacing Electrophysiology, 1996; Eckberg, 1997; Mujica-Parodi et al., 2009). Because PSD is a linear technique, it fails to account for non-linear properties of HR control. To overcome the inability of the PSD approach to separate the dynamics pertaining to the ANS, we have previously developed and validated a novel mathematical technique, the Principal Dynamic Mode analysis (PDM), which is able to extract and separate the sympathetic and parasympathetic dynamics (Zhong et al., 2004). The PDM was shown to provide responses that were more in agreement with the expected physiological responses when compared to the PSD in a human study involving pharmacological blockades (Zhong et al., 2006; Bai et al., 2009; Mujica-Parodi et al., 2009). The PDM has also been applied to renal blood pressure and flow data to better characterize renal autoregulatory mechanisms, further supporting the general applicability of the method (Chon et al., 1994, 1998). For another non-linear measure, we used approximate entropy (ApEn) (Pincus, 1991). ApEn is a statistical index for quantifying the complexity of a time series signal, and its use is appealing because quantitative insight about the regularity (orderliness) of the underlying patterns within the data signal can be obtained. ApEn measures the likelihood that runs of patterns that exhibit regularity over a certain duration of data also exhibit similar regular patterns over the next incremental duration of data. In other words, it calculates the conditional probability that two sequences with a specified window length will remain similar from one point to the next. Thus, signals associated with a high degree of regularity correspond to a more ordered system, and as such, exhibit lower ApEn values, while signals associated with irregularity and greater randomness correspond to a less ordered (or more disordered) system, and as such, exhibit higher ApEn values.

Thus, in this study, we applied the non-linear methods PDM and ApEn to assess the ANS dynamics during SCUBA diving at different depths and gas mixtures consisting of air, nitrox and trimix. The dive profile was designed to ensure divers maintained instructed depths for pre-determined durations. Only the stable portions of ECG recordings at each depth were extracted, which allowed us to estimate the depth effect on the ANS dynamics during diving. Additionally, effects of different breathing gases on the ANS were also examined. As far as we are aware, this is one of the first studies to collect ECG data from SCUBA divers at various depths using a variety of gas mixtures.

## Materials and methods

### Experiment protocol

The study protocol was approved by Worcester Polytechnic Institute's Institutional Review Board in compliance with all applicable Federal regulations governing the protection of human subjects. All subjects gave written informed consent in accordance with the Declaration of Helsinki.

Twenty-four experienced SCUBA and rebreather divers participated in this study. Of these, 16 were SCUBA divers (open-circuit divers) and 8 were rebreather divers. Divers were assigned to perform dives at different depths and breathing gases. Each diver participated in multiple dives over 5 days. SCUBA dives (consisting of both open-circuit divers and rebreathers) were carried out at 5 different depths (33, 66, 99, 150, and 200 ft; equivalent to 10.05, 20.10, 30.17, 45.72, and 60.96 m, respectively). The dives at 33 and 66 feet were carried out on the first day in the morning and afternoon, respectively. Dives to 99, 150, and 200 ft. were carried out on the next 3 days, one depth per day. The 5th day was for dives that required additional data. Three different breathing gases (air, nitrox, and trimix) were employed. Table 1 summarizes in detail the dive protocols for all depths. Note that air contains approximately 21% oxygen and 79% nitrogen. Nitrox contains 36% oxygen and 64% nitrogen. Trimix contains 10% oxygen, 50% helium, and 40% nitrogen.

**Table 1:** Summary of dive information of various depths and durations.

**Depth**	**Bottom duration**	**Breathing gas**	**Water temperature(°C)**	**# of divers**	**Age**	**Weight (kg)**
33 ft. (10.05 m)	30 min	Air	14.96 ± 0.82	11	42.53 ± 2.17	83.50 ± 3.16
66 ft. (20.1 m)	30 min	Air	12.78 ± 0.57	11	41.13 ± 2.03	83.28 ± 2.85
99 ft. (30.17 m)	15 min	Air	14.74 ± 0.69	13	42.31 ± 2.57	85.17 ± 3.31
99 ft. (30.17 m)	15 min	Nitrox	14.49 ± 0.77	14	41.92 ± 2.41	84.43 ± 3.15
150 ft. (45.72 m)	15 min	Trimix	12.73 ± 0.43	12	43.08 ± 1.83	89.77 ± 2.19
200 ft. (60.96 m)	10 min	Trimix	9.75 ± 0.36	10	40.60 ± 2.40	90.54 ± 2.21

*Water temperature is the average temperature at the bottom recorded by a diving computer. Air contains 21% oxygen and 79% nitrogen. Nitrox contains 36% oxygen and 64% nitrogen. Trimix contains 10% oxygen, 50% helium and 40% nitrogen*.

Prior to each dive, divers donned a five lead digital Holter ECG monitor (RZ153+, Rozinn Electronics, Cleveland, OH). ECG electrodes were securely placed on specific locations (as specified by the Holter vendor) of a diver's body with adhesive tape. Each diver wore a thermal undergarment and a dry diving suit (Bare, Langley, British Columbia) which insulated the Holter monitor from sea water. A diving data logger (GEO, Oceanic, San Leandro, CA) was used to record each diver's dive profile including the dive duration, depth and water temperature.

After entering the water, the divers floated on the surface for 10 min with minimal movement, in the supine position, with their faces out of water; ECG data recorded during this phase is considered the baseline. Once the baseline ECG recordings had been completed, divers descended to an assigned depth; when the depth was reached, divers remained in a horizontal body position with minimal movement for the pre-determined duration. The depth and time duration information for each dive is summarized in Table 1.

Of the 10 min ECG recording at the surface for each dive, the last 5 min segment of data was used as the baseline value. The stable portions of ECG recordings at depth, also referred to as the bottom time, were divided into 5 min segments for HRV analysis. For the 33 and 66 ft. dives, the bottom time duration was as long as half an hour. Thus, we used data from these two depths to investigate diving time effects on the ANS. To understand physiological changes at different diving depths, the 3rd 5 min time segment of the bottom time at 33, 66, and 99 ft. and the 2nd 5 min time segment at 150 and 200 ft. were used to perform our analysis. We chose the 2nd 5 min time segment for the 150 and 200 ft. dives instead of the 3rd 5 min data because divers already had a longer time to equilibrate at these two deep dives than the shallow dives. For the breathing gases used, the 33, 66, and 99 ft. dives had the divers breathing air (nitrox was also used at 99 ft), but the 150 and 200 ft. dives used trimix gases. Finally, we studied the effect of different breathing gases on diving by comparing dives using either air or nitrox at 99 ft.

### ECG analysis

ECG measurements were collected by a Holter monitor with a sampling rate of 180 Hz. R waves in the ECG recordings were detected using automated software developed for the Rozinn monitor (Holter for Windows+). Any incorrectly recognized R waves were manually corrected. Once heart beat timing was determined, an instantaneous HR signal was created at a sampling rate of 4 Hz using the technique described in reference (Berger et al., 1986). HR signals were then down sampled to 1 Hz with the mean and low-frequency trends removed. Signal segments containing 300 data points, which corresponds to 5 min, were used for both the PDM and PSD analyses.

### Analysis of HR using principal dynamic modes

The PDM is a non-linear method which is designed to extract only the principal dynamic components of the signal via eigen decomposition. The PDMs are calculated using the Volterra-Wiener kernels based on expansion of Laguerre polynomials (Marmarelis, 1993). Among all possible choices of expansion bases, some require the minimum number of basis functions to achieve a given mean-square approximation of the system output. This minimum set of basis functions is termed the PDMs of the non-linear system. PDM specifically accounts for the inherent non-linear dynamics of HR control, which the PSD does not. A minimum set of basis functions is determined using a method widely known as principal component analysis, in which the dominant eigenvectors and eigenvalues are retained as they relate more closely to the true characteristics of the signal, and non-dominant eigenvectors and eigenvalues are considered to represent noise or non-essential characteristics. Thus, principal component analysis separates only the essential dynamic characteristics from a signal that is likely to be corrupted by noise and non-system related dynamics. In the case of the HR signal, the dominant eigenvectors and eigenvalues should reflect the dynamics of the sympathetic and parasympathetic systems. We have modified the PDM technique to be used with even a single output signal of HRV data, whereas the original PDM required both input and output data. A detailed summary of the procedure has been presented in our previous study (Zhong et al., 2004), and comparisons between the PDM and PSD have been made using the same data (Zhong et al., 2004, 2006).

While the PDM is a time-domain representation, we convert it to the frequency domain via the Fast Fourier Transform (FFT) to facilitate interpretation of the two ANS activities, as they are usually illustrated in the frequency domain. Therefore, hereafter we will describe the PDMs' dynamic characteristics in the frequency domain. For this study, we used 8 Laguerre functions with a memory length of 60. The detailed steps involved in the calculation of PDMs as well as determining the Laguerre functions and the memory lengths have been previously described (Zhong et al., 2004). Henceforth, the derived PDMs' two main dynamics will be termed the PDMsymp and PDMpara. The frequency bands of PDMsymp and PDMpara are within the same bands of LF and HF derived from PSD.

### Power spectral density

Power spectral densities of HR data were calculated using the method of the Welch periodogram (Matlab 7.0, Natick, MA). A 128**-**point FFT (frequency resolution of 0.0078 Hz) with the Hanning window and 50% overlapping segments were used. We denote the LF and HF bands as 0.04–0.15 Hz and 0.15–0.4 Hz, respectively, as there was a clear separation of spectral powers between these frequency bands and they are in keeping with the recommendation from the HRV task forces (Task Force of the European Society of Cardiology and the North American Society of Pacing Electrophysiology, 1996). Henceforth, the PSD's derived sympathetic and parasympathetic dynamics will be named the LF and HF, respectively.

### Time-domain parameters

The mean HR, root-mean square of the successive difference (RMSSD) of the RR intervals and the standard deviation of normal-to-normal R waves (SDNN) of the HR were calculated. RMSSD mainly reflects the parasympathetic tone and SDNN is an indicator of overall ANS activity (Task Force of the European Society of Cardiology and the North American Society of Pacing Electrophysiology, 1996).

### Respiratory rate estimation from ECG

The ECG has been used in many different ways to quantify effects of breathing. The most well-known respiration-related effect is its influence on the variability of HR (respiratory sinus arrhythmia). In this regard, there has been a profusion of research on so-called ECG-derived respiratory activity (EDR) measures, with a multitude of signal processing algorithms available to derive respiratory-induced modulations from both multi-lead and single-lead ECG signals. The simplest single-lead ECG algorithms use the amplitude modulations in or variations in area under the QRS complex or T-wave (Bailón et al., 2006). For our approach we use a time-frequency method known as the complex demodulation method to derive respiratory rates. Details of the algorithm have been presented (Wang et al., 2006) but the main idea is to extract frequency modulation time series at the HR frequency for all time points. This is in keeping with the well-known observation that breathing has a frequency modulation effect on the HR. We then simply take the power spectrum of the frequency modulation time series. The frequency at which the highest peak occurs within a specified breathing frequency range of 0.15–0.7 Hz is the detected breathing rate (Dash et al., 2010).

### Approximate entropy (ApEn)

In this section we provide a step-by-step procedure for calculation of the ApEn of HR.

Given a signal x(n) = x(1), x(2),…, x(N), where *N* is the total number of data points, the ApEn algorithm implemented in our study can be summarized as follows:

(a) generate *m*-vectors from the original time series for *i* = 1,N−m+1, *X(1)* to *X(N*−*m*+*1)*, defined by *X(i)* = [*x(i)*,*x(i*+1),…*x(i*+*m*−1)]
(b) define the difference between two such vectors, *X(i)* and *X(j)*, as $d_{m}[X(i),X(j)=\underset{k=0,m-1}{\max}[\left|x(i+k)-x(j+k)\right|]$, which is the maximum absolute difference of their respective scalar components
(c) define the function $C_{i}^{m}\left(r\right)$ for *i* = 1, N−m+1 as
  $$C_{i}^{m}(r)=V^{m}(i)/(N-m+1)$$
where $V^{m}(i)=no.ofd_{m}[X(i),X(j)]<r$
(d) obtain the natural logarithmof each $C_{i}^{m}(r)$ and average them over *i* as described in step (c)
  $$\phi^{m}(r)={\left(N-m+1\right)}^{-1}\sum_{i=1}^{N-m+1}\ln(C_{i}^{m}(r))$$
(e) increase the dimension to *m*+1, and repeat steps (a)-(d)
(f) calculate the ApEn value for a finite data length of *N* as follows:
  $$\begin{array}{l} ApEn\left(m,r,N\right)=\phi^{m}\left(r\right)-\phi^{m+1}\left(r\right) \end{array}$$

The algorithm described above require *a priori* specification of the parameters *m* and *r*, and although the exact values for these parameters are unknown, parameter selection criteria have been established for signals with slow dynamics (e.g., HR, hormone secretion). In this case, for computation of ApEn, we followed the recommendation that *m* be set to 1 or 2 and that *r* be set between 0.1 and 0.25 × SD of the data for data lengths of *N* ranging from 100 to 5,000 data points (Pincus, 1991; Pincus and Goldberger, 1994).

### Statistical analysis

Data are represented as mean ± standard error. The normality of the indices was tested using the one-sample Kolmogorov-Smirnov test (Massey, 1951; Miller, 1956; Wang et al., 2003). If non-normality was found, non-parametric statistical techniques were used. For the investigation of varying dive times, analysis of repeated measurements was used to compare parameters at different time segments. In the different depths analysis, parameter values from the second or third 5 min time segment in each dive depth were regarded as the bottom value and compared to the baseline by a comparison of the two groups (e.g., baseline vs. each depth). To compare among different depths, the difference between the baseline and bottom was first taken for the dives at each depth, and then analysis of repeated measurements was employed to compare these diving-induced alterations at different depths. For the comparison of air and nitrox gas dives, the difference between the baseline and bottom was also taken and the two groups were compared.

For the analysis of repeated measurements in normally-distributed data, the one-way analysis of variance (ANOVA) was performed to test for significant differences among dive times. When significant F-ratios were obtained by ANOVA, Student-Newman-Keuls tests were used for multiple comparisons. When data were non-normally distributed, we used the Dunn's test, a non-parametric multiple comparisons procedure based on rank sums, used as a “*post-hoc*” procedure following rejection of a Kruskal–Wallis test to compare parameters at different time segments. This technique is a non-parametric analog to multiple pairwise *t*-tests following rejection of an ANOVA null hypothesis (Cardillo, 2006). For the comparison of two groups, we used the *t*-test if data were normally distributed, or a two-sided Wilcoxon rank sum test if non-normality was found (Gibbons and Chakraborti, 2011). A *p* < 0.05 was considered significant.

## Results

### Time effect of diving

**Figure 1:**
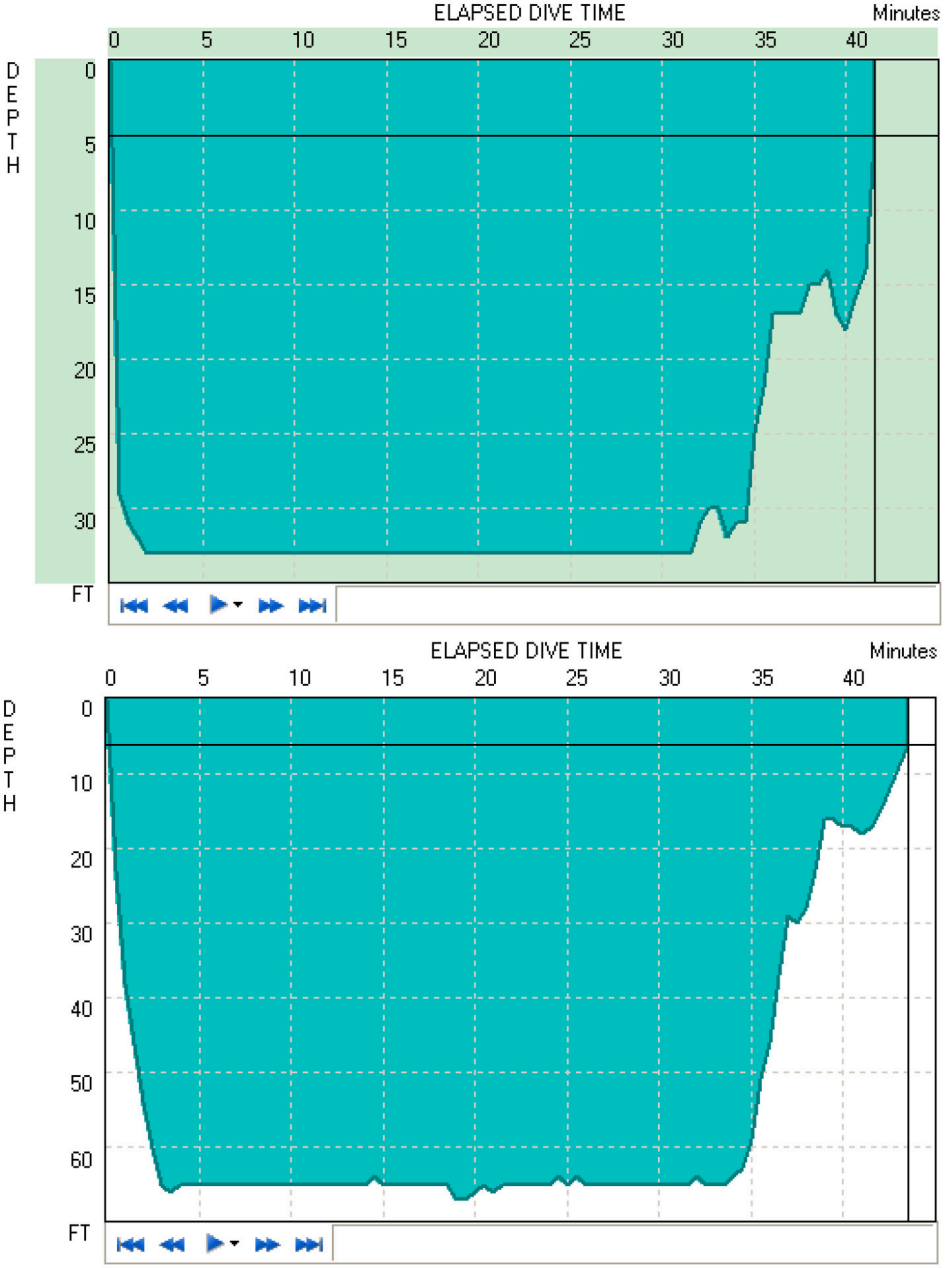
Typical dive profiles of the 33 ft. **(Top)** and 66 ft. **(Bottom)** dives, respectively.

Figure 1 shows two typical diving profiles to 33 and 66 ft. As shown in the figure, these divers were able to maintain the desired depth for the entire duration. However, only 11 of 16 open-circuit SCUBA divers were able to maintain the instructed depth for 30 min to obtain data recordings. Thus, the average of only 11 subjects' data was used to study the time and depth effects of diving.

All indices measured for the different diving times were found to be normally distributed. For the 33 ft. dive, HR during the bottom period was significantly lower (*p* < 0.05) than the baseline, as shown in Table 2. The PDMpara significantly increased throughout the bottom period when compared to the baseline. The increase of RMSSD was significant after diving for 10 min. There was also an increase of SDNN at 33 ft. after diving for 20 min. However, the PDMsymp dynamics, as well as LF and HF of PSD did not show any significant alterations during the dive, although there was a trend of increase in the LF and HF values of the PSD.

**Table 2:** HRV parameters during 33 ft. dive.

	**Baseline**	**Dive5**	**Dive10**	**Dive15**	**Dive20**	**Dive25**	**Dive30**
HR	99.51 ± 2.04	94.25 ± 3.80^*^	89.16 ± 2.99^*^	85.98 ± 2.69^*^	84.29 ± 2.31^*^	83.66 ± 2.27^*^	84.17 ± 1.99^*^
PDMsymp	0.145 ± 0.009	0.149 ± 0.008	0.133 ± 0.011	0.148 ± 0.016	0.158 ± 0.010	0.144 ± 0.009	0.148 ± 0.010
PDMpara	0.178 ± 0.008	0.233 ± 0.022^*^	0.212 ± 0.020^*^	0.225 ± 0.028^*^	0.234 ± 0.023^*^	0.245 ± 0.018^*^	0.238 ± 0.019^*^
RMSSD	14.70 ± 1.88	19.61 ± 7.36	20.01 ± 2.07	23.25 ± 2.80^*^	25.64 ± 3.21^*^	29.73 ± 4.01^*^	26.59 ± 2.82^*^
SDNN	33.75 ± 4.41	43.02 ± 2.22	40.71 ± 3.64	41.80 ± 3.48	46.24 ± 3.88	48.76 ± 3.53^*^	52.69 ± 3.84^*^
LF	12.82 ± 2.74	16.92 ± 3.22	15.14 ± 3.04	13.42 ± 2.29	14.93 ± 3.16	14.03 ± 2.44	17.11 ± 3.42
HF	2.75 ± 0.49	4.24 ± 1.13	2.72 ± 3.79	3.60 ± 1.22	3.20 ± 0.86	5.48 ± 1.56	4.59 ± 1.30
LF/HF	5.58 ± 0.88	6.33 ± 1.24	8.15 ± 2.01	8.41 ± 2.45	6.57 ± 1.54	5.50 ± 1.72	6.00 ± 1.51
ApEn	1.01 ± 0.03	0.96 ± 0.05	0.99 ± 0.03	0.98 ± 0.05	1.00 ± 0.03	1.00 ± 0.03	0.94 ± 0.02

Dive5 represents first 5 min segment of dive, and dive10 represents from 5 to 10th min, and so on.

* *P < 0.05 when compared with baseline*.

The results of the 66 ft. dive are summarized in Table 3. The HR of the baseline was higher (*p* < 0.05) than that of the bottom period. The PDMpara increased significantly throughout the dive. Both RMSSD and SDNN values increased significantly when compared to the baseline. The HF significantly increased compared to the baseline when time at the bottom lasted for more than 15 min. In the 66 ft. dive, there was only a slight non-significant increase in the LF and the PDMsymp dynamics during the bottom stage.

**Table 3:** HRV parameters during 66 ft. dive.

	**Baseline**	**Dive5**	**Dive10**	**Dive15**	**Dive20**	**Dive25**	**Dive30**
HR	101.41 ± 2.93	95.11 ± 3.87	87.05 ± 3.57^*^	84.2 ± 3.12^*^	81.72 ± 2.61^*^	80.29 ± 2.48^*^	81.92 ± 2.34^*^
PDMsymp	0.130 ± 0.009	0.162 ± 0.013	0.163 ± 0.016	0.156 ± 0.017	0.152 ± 0.015	0.164 ± 0.024	0.175 ± 0.011
PDMpara	0.194 ± 0.020	0.258 ± 0.030^*^	0.221 ± 0.019	0.268 ± 0.034^*^	0.279 ± 0.032^*^	0.285 ± 0.023^*^	0.257 ± 0.023^*^
RMSSD	13.44 ± 1.94	29.21 ± 6.99^*^	30.86 ± 7.72^*^	38.47 ± 8.41^*^	43.76 ± 8.78^*^	47.36 ± 8.99^*^	44.12 ± 7.80^*^
SDNN	36.74 ± 5.19	55.80 ± 8.90^*^	53.14 ± 9.35^*^	54.79 ± 9.03^*^	64.10 ± 9.79^*^	67.94 ± 10.27^*^	73.17 ± 9.93^*^
LF	11.32 ± 3.43	23.74 ± 3.29	27.46 ± 10.98	19.98 ± 7.74	24.24 ± 8.65	25.37 ± 6.94	26.72 ± 7.81
HF	2.00 ± 0.44	6.96 ± 0.88	7.18 ± 2.51	7.33 ± 2.53	8.83 ± 3.03^*^	9.87 ± 2.72^*^	11.02 ± 3.09^*^
LF/HF	5.64 ± 0.82	4.43 ± 0.74	6.27 ± 1.88	4.29 ± 1.16	3.54 ± 0.80	3.92 ± 1.01	3.10 ± 0.69
ApEn	0.99 ± 0.06	0.98 ± 0.04	0.98 ± 0.04	0.97 ± 0.05	1.01 ± 0.03	0.98 ± 0.03	0.96 ± 0.04

* *P < 0.05 when compared with baseline*.

In both dives (33 and 66 ft.), the parasympathetic parameters, including the HF, RMSSD and the PDMpara, showed an insignificant trend of increase from the beginning of the dives, until they reached a peak value between the 20 and 25th min of dives, and then an insignificant decrease in the last segment of the bottom time of the dive. However, the HR decreased as the dive continued and reached its lowest point at the 5th 5 min segment, and then increased at the 6th time segment of the bottom time. Meanwhile, the PDMsymp dynamics and the LF of PSD did not show any significant trend throughout these two dives.

### Depth comparison

**Figure 2:**
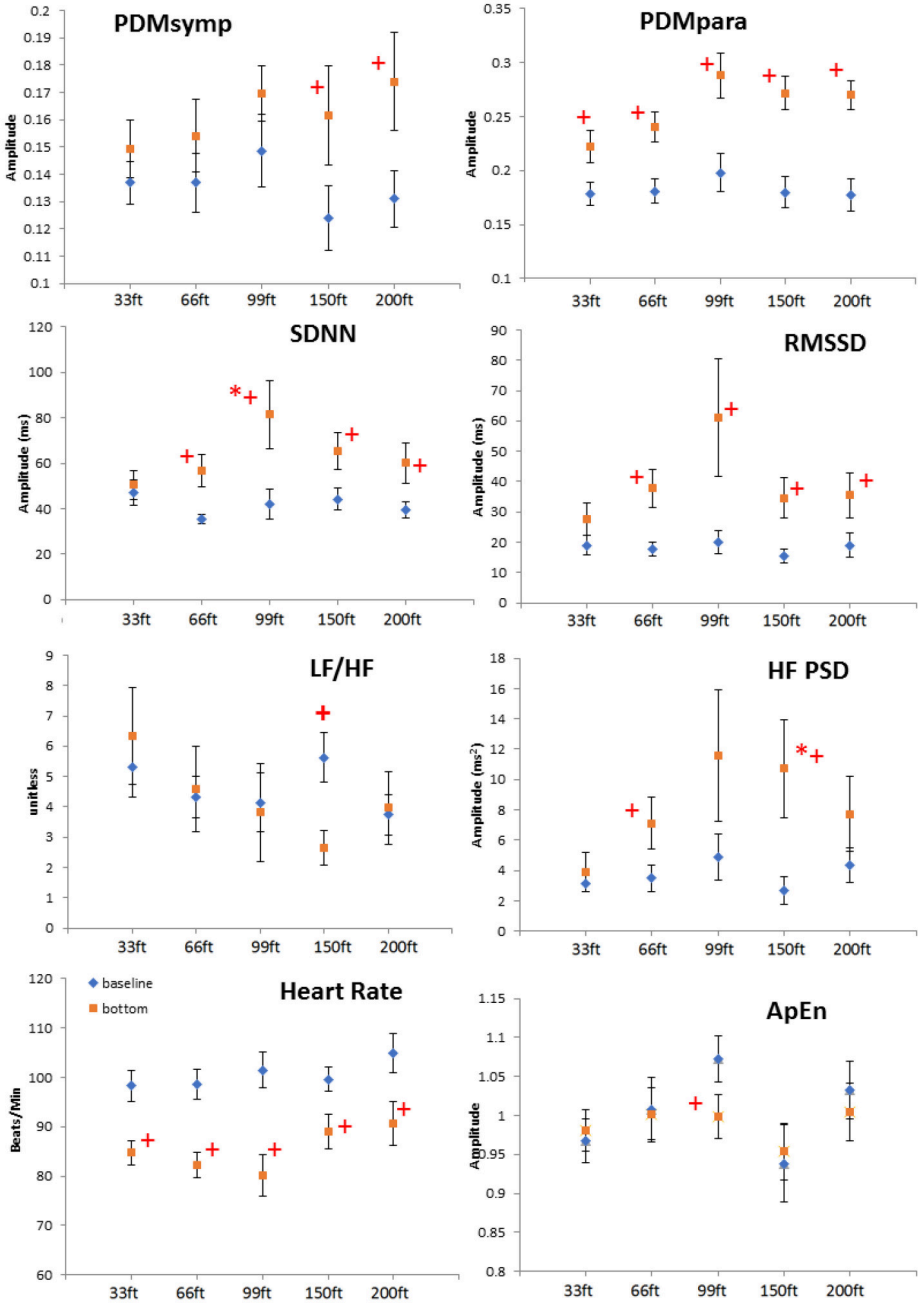
Depth comparison of HRV parameters. The line marked with diamonds represents baseline; the square line is bottom condition. + means *p* < 0.05 when comparing the bottom to its corresponding baseline by paired *t*-test. *denotes *p* < 0.05 when comparing to the 33 ft. condition. Error bars denote standard error.

All indices were found normally distributed, except for LF. For all dives at different depths, the HR at the bottom showed a significant decrease when compared to the baseline (Figure 2). The PDMpara increased significantly at the bottom. The HF also increased at all depths when compared to the baseline, but this increase was significant only at 66 and 150 ft. Both the LF and the PDMsymp dynamics at the bottom increased when compared to the baseline. Additionally, the increase of the PDMsymp dynamics was significant at 150 and 200 ft. The RMSSD and SDNN (which reflects the overall cardiac ANS tone) indices were significantly increased for 66, 99, 150, and 200 ft. The LF/HF ratio was significantly increased only at 150 ft. ApEn was significantly decreased at 99 ft.

When comparing different depths, only the increase of the SDNN and the HF during the 99 and 150 ft. dives, respectively, were significantly higher than during the 33 ft. dive. However, the other parameters did not show a significant difference at different depths. It can be noted that the bottom values of the RMSSD, HF and the PDMpara reached their maximum during the 99 ft. dive (Figure 2). As the diving depth increased, the sympathetic dynamics showed a trend of concomitant increase.

### Respiratory rates

We have used our recently-developed algorithm to accurately estimate breathing rates directly from ECG signals without using a dedicated respiratory rate sensor (Chon et al., 2009; Dash et al., 2010). Using our respiratory rate detection algorithm, the average respiratory rates were found not to be significantly different between 99 ft. (0.246 ± 0.06 Hz) to 150 ft. (0.281 ± 0.10 Hz), or 200 ft. (0.286 ± 0.08 Hz).

### Effect of breathing gases

**Figure 3:**
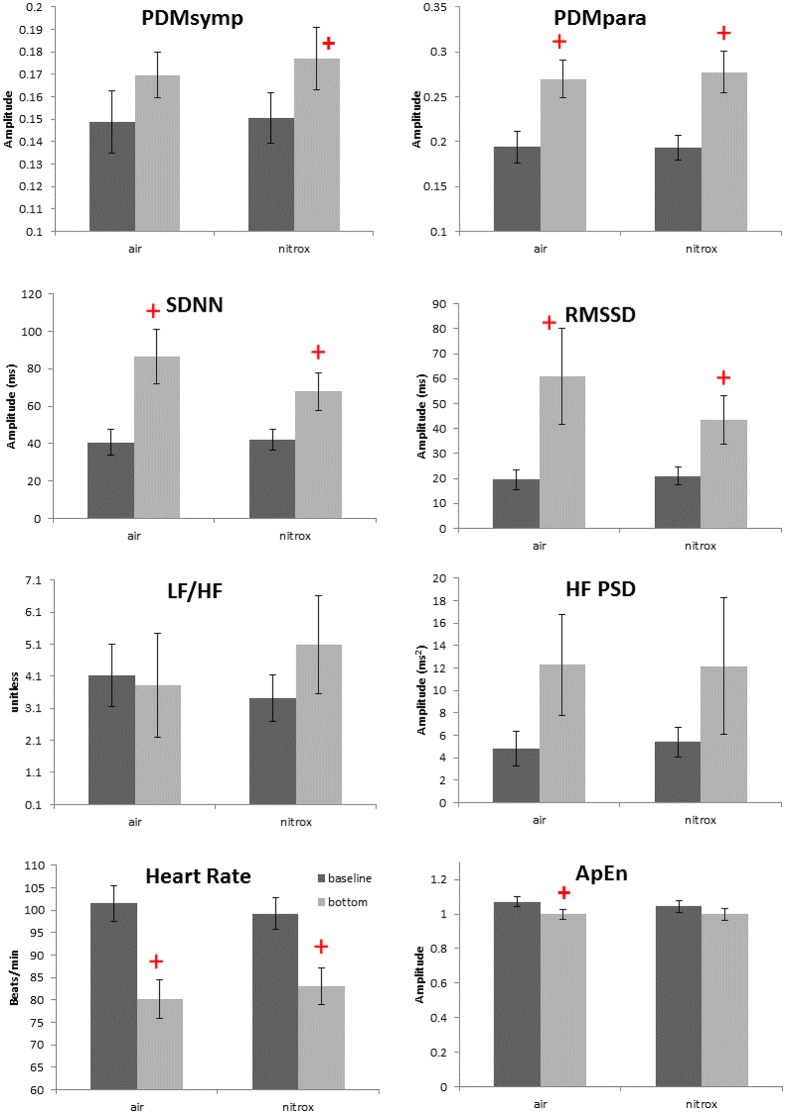
Effects of breathing gases on HRV parameters. Darker bars represent the baseline and the gray bars represent the bottom condition (99 ft.). + denotes *p* < 0.05 when comparing the bottom to the corresponding baseline. Error bars denote standard error.

Non-normality was found only in the HF index. For the 99 ft. dives, both air (*N* = 13 subjects) and nitrox (*N* = 14 subjects) gases were used. Compared to baseline, these two dives induced a decrease in the HR and an increase in the SDNN, RMSSD and the PDMpara, which is similar to other dive depths. Also, the PDMsymp were only increased compared to baseline for nitrox, and ApEn was significantly reduced compared to baseline for the air. However, as shown in Figure 3, no significant differences were found in these parameters between the air and nitrox gas dives. The bottom values of these parameters were nearly at the same level for the two gases. No significant differences between 99 ft. and baseline were found for the LF/HF ratio.

## Discussion

In all dives, a decrease in the HR and dominance of the parasympathetic regulation compared to the baseline were observed despite different diving depths and breathing gases. For 33 and 66 ft. dives, the PDMpara increased as the diving duration increased. As the diving depth increased to 150 and 200 ft., divers may have experienced more diving stress which can result in increased sympathetic dynamics as compared to baseline values. The PDMpara at both 150 and 200 ft. showed a decreasing trend when compared to those at 99 ft. These results at 150 and 200 ft. dives are in agreement with a prolonged hyperbaric saturation diving study which showed elevated sympathetic nervous activities and reduced parasympathetic nervous activities (Hirayanagi et al., 2003). To our knowledge, ours is the first study that evaluates the effects of time duration, different depths, and breathing gases in SCUBA dives. In the two other published SCUBA studies with the aim of examining the ANS during diving, one was carried out in a swimming pool (Schipke and Pelzer, 2001) and the other involved recreational dives all at shallow depths of 66 ft. (Chouchou et al., 2009).

The baseline values were collected when the divers floated on the water surface without face immersion. The reason for this is that we observed a heavy equipment load and a dry suit with thermal protection added extra burden to divers and resulted in a much higher HR (around 110–120 bpm at surface) than without them. Floating was done to try to minimize the workload burden seen at the surface and equalize the workload burden between surface (baseline) and bottom stages, as divers donned the same equipment load and dry suit for both depths. It is possible that decreases in HR and increases in the parasympathetic activity indices observed when the baseline values are compared against their values at the various depths resulted from relaxation at the water surface phase of the recordings. However, we have tried to minimize these effects by collecting data for 10 min and analyzing only the last 5 min portion of the data during water surface recordings. Moreover, the subjects were all experienced divers, hence, the workload stress at the surface should have been minimized by the time the 10 min baseline recordings at the water surface were taken.

Our dive experiments were performed in relatively low water temperature (17.8°C at the surface). A previous study has shown that body immersion in water of 14°C or less induces an increase of HR and metabolic activity (Srámek et al., 2000). Though the bottom water temperature in the present study was near 14°C (Table 1), we observed decreased HR during the bottom stage. Our data suggest that, with adequate thermal protection from the undergarment and dry suit and the relatively short dive duration (Nuckols et al., 2012) thermal adjustment, if any, was not a main factor influencing the HR activity in this study. Thus, the physiological changes between the baseline and diving are most likely induced by a diving reflex, increased ambient pressure, high density of breathing gas and psychological stress.

### Time effect

For the 33 and 66 ft. dives, the decreased HR and increased parasympathetic parameters (HF, RMSSD, and the PDMpara) during diving illustrate the dominance of the parasympathetic system (Tables 2, 3). During the entire bottom duration, the above noted parasympathetic parameters showed an increasing trend until the 25th min of bottom time and then decreased during the last 5 min segment of the dives albeit this increasing trend was found to be non-significant. We believe the non-significant decrease in the last segment may partly be due to the fact that some divers did not stay at the bottom as instructed but began ascending to the surface. In general, we found that the cardiac ANS dynamics gradually changed to adapt to the underwater environment but our time duration of 30 min was not enough to reach a steady state during SCUBA diving. This time effect certainly should be considered in any diving studies. Thus, for the depth comparison of this study, we used the 3rd 5 min time segment of the bottom time at 33, 66, and 99 ft. and the 2nd 5 min time segment at 150 and 200 ft. to perform the comparison among varying depths, because it took approximately 5 more min to reach the bottom stage with deeper dives. The rationale is that divers already had longer time to equilibrate at these two deep dives than the shallow dives, hence, our use of the 2nd 5 min time segment instead of the 3rd 5 min time segment at 150 and 200 ft. dives.

For two of the depths, 33 and 66 ft., the sympathetic dynamics and LF of PSD during diving were not significantly different when compared to the baseline and did not show a clear trend throughout the bottom time. This suggests that the sympathetic regulation was not changed in these shallow dives and the alterations of SDNN were mainly induced by the parasympathetic system. Our results showing increased parasympathetic nervous dynamics and a non-significant change in the sympathetic nervous dynamics are in agreement with two other SCUBA studies involving 13 ft. (Schipke and Pelzer, 2001) and 66 ft. (Chouchou et al., 2009) depths. Both LF/HF and ApEn maintained stable values, exhibiting no significant differences during the 30 min dive.

### Depth comparison

At all depths, HR at the bottom decreased compared to the baseline. Moreover, the parasympathetic dynamics showed a significant increase. In the 150 ft. dive, the difference in the HF PSD index (linked to parasympathetic control) between the baseline and bottom was significantly higher than for the 33 ft. dive (Figure 2). This suggests an enhanced cardiac parasympathetic tone of HRV in deep dives compared to the 33 ft. dive. The values of the parasympathetic parameters reached a peak at 99 ft., and then slightly decreased for the 150- and 200 ft. dives. At different depths, the sympathetic dynamics increased compared to the baseline. But this increase was significant only for the 150 and 200 ft. dives, indicating a greater sympathetic activity in these two dives compared to shallower dives. The only significant difference in the sympatho-vagal balance, assessed by LF/HF ratio, was in the 150 ft. dive. Also, a significant decrease in ApEn was found only at 99 ft. While respiration rate was not controlled at a constant frequency at these deep depths, we do not believe that the increased sympathetic tone resulted from decreased breathing rates; this would result in the spectral power spilling over to the LF range. Using our respiratory rate detection algorithm (Chon et al., 2009; Dash et al., 2010), the average respiratory rates are found not to be significantly different between 99 ft. (0.246 ± 0.06 Hz) to 150 ft. (0.281 ± 0.10 Hz) or 200 ft. (0.286 ± 0.08 Hz).

After face immersion, the diving reflex and water pressure induce bradycardia and the activation of the parasympathetic system (Flook, 1987; Miwa et al., 1997). As the depth increases, increased gas density and water pressure may further increase parasympathetic tone and decrease HR (Hong et al., 2011; Pendergast et al., 2015; Berry et al., 2017). In our study, this phenomenon was observed as the parasympathetic tone increased with increasing depth up to 99 ft. However, for the 150 and 200 ft. dives, there was a trend of increase in the sympathetic activity and a decrease in the parasympathetic regulation were observed when compared to the 99 ft. dive. This response is to be expected since diving in deeper and colder water with greater current strength will cause substantial stress on even the most experienced SCUBA divers.

Previous studies have shown that mental stress causes the sympatho-vagal balance to be tilted toward the sympathetic system (Pagani et al., 1991; Sloan et al., 1996). More importantly, mental stress can also attenuate the amplitude of the diving reflex (Ross and Steptoe, 1980). However, we believe the parasympathetic tone was still dominant in the 150 and 200 ft. dives because bradycardia was present in these two dives. Indeed, these observations are reflected in Figure 2 where there were significant increases in both the sympathetic (only found with PDMsymp and not LF) and the parasympathetic (HF, RMSSD, and PDMpara) at both 150 and 200 ft. when compared to baseline values.

### Effects of breathing gas

As the ambient pressure increases during diving, the density and partial pressure of gas components in the breathing gas mixture also increase. Among different gas components, a high partial pressure of oxygen can induce bradycardia, decrease sympathetic activity, and increase parasympathetic tone. The mechanism of hyperoxic bradycardia is under debate and remains unresolved. The possible reason could be that the hyperoxia induces arteriolar vasoconstriction, or reduces input stimuli to the peripheral or central chemoreceptors (Shida and Lin, 1981; Seals et al., 1991). Thus, in this study, the increased partial pressure of oxygen may have contributed to the diving-induced bradycardia and the increase of the parasympathetic tone.

We assessed the ANS activity while breathing either air or nitrox during 99 ft. dives. However, no difference was found in HR and ANS activity between air and nitrox dives. In a normobaric condition, significant decreases in HR and increases in parasympathetic activity as measured by the HF from baseline were observed when the proportion of oxygen in the breathing gas was at least 70% (Shibata et al., 2005). Another study also showed that cardiac parasympathetic tone, though greater than during normobaric air breathing, was not significantly different between exposures with normobaric 100% oxygen and hyperbaric air and 100% oxygen up to 2.5 atmospheres absolute (Lund et al., 1999). These studies and our results suggest that the greater oxygen content of nitrox (36%) compared to air (21%) is not enough to induce significant differences in HRV between the two breathing mixes at 99 ft. Another possibility is that because the diving reflex and water pressure have already increased the parasympathetic tone to a very high level, hyperoxia does not further enhance the parasympathetic activity. Thus, changes in the oxygen concentration in addition to the immersion adjustments are unable to induce significant ANS alterations. The main factors inducing the ANS changes among varying depths were most likely different pressures and mental stress, and not different gas mixtures.

### Limitations of the study

This work involved the significant involvement and commitment of various SCUBA and rebreather divers. While we made our best attempt to recruit more SCUBA and rebreather divers, due to a limited budget and pool of experienced divers, the sample size was relatively small. This may explain why we did not see any significant changes between air and nitrox breathing for the parameters examined in this work, for example.

Additionally, variation in temperature with depth (which may have had a minor effect as it only offered by 3.8°C temperature difference and thermal protection was used and no core body temps were taken) could not be controlled which may have affected some of the results presented in this work. However, all divers donned dry suits with thermal undergarments to minimize the effect of temperature variation at varying depths, hence, we believe this effect may have been minimal.

In conclusion, our study found a predominance of parasympathetic activity in SCUBA diving at all depths, including the deep dives never studied before. This finding, for shallower dives, is consistent with previous diving studies (Schipke and Pelzer, 2001; Chouchou et al., 2009) as well as hyperbaric chamber studies (Lund et al., 1999, 2000) albeit the latter involved hyperoxia (100% O_2_ up to 3 ATA). In the deep 150 and 200 ft. dives, we also found a significant increase in the sympathetic nervous dynamics in addition to the elevated parasympathetic dynamics when compared to the baseline condition. We have also found that it takes considerable time for the ANS to reach steady state in the underwater environment. As the diving depth increases, mental stress, temperature, workload or the increase in the partial pressure of the gasses may become important factors influencing the ANS activities. Nitrox and air did not have different effects on the ANS in the 99 ft. dive. Finally, we found that the ANS dynamics evaluated by the PDM provided more physiologically consistent results than those obtained from the PSD. This statement is also supported by our previous study which showed more consistent and better detectability of neurological decompression sickness in swine using PDMsymp and PDMpara which were significantly depressed (Bai et al., 2009). Hence, using the PDM method to monitor the ANS status provides a better assessment of physiological changes during diving than using the PSD. The PDM method could potentially be used as a tool for predicting and avoiding hazardous diving conditions such as decompression sickness.

## Author contributions

YN: performed the data analysis and wrote a draft of the paper; HP-Q: conducted the statistical analysis and edited the paper; YB: performed the experiments, data analysis, and edited the paper; JW: performed the experiments, analysis of the results, and edited the paper; JF and PB: conducted analysis of the results and edited the paper; KC: conceptualized the study and edited the final version of the paper.

### Conflict of interest statement

The authors declare that the research was conducted in the absence of any commercial or financial relationships that could be construed as a potential conflict of interest.
